# The Ca_V_β Subunit Protects the I-II Loop of the Voltage-gated Calcium Channel Ca_V_2.2 from Proteasomal Degradation but Not Oligoubiquitination*

**DOI:** 10.1074/jbc.M116.737270

**Published:** 2016-08-03

**Authors:** Karen M. Page, Simon W. Rothwell, Annette C. Dolphin

**Affiliations:** From the Department of Neuroscience, Physiology and Pharmacology, University College London, Gower St., London WC1E 6BT, United Kingdom

**Keywords:** calcium channel, confocal microscopy, protein degradation, trafficking, ubiquitin, ubiquitylation (ubiquitination), Western blot

## Abstract

Ca_V_β subunits interact with the voltage-gated calcium channel Ca_V_2.2 on a site in the intracellular loop between domains I and II (the I-II loop). This interaction influences the biophysical properties of the channel and leads to an increase in its trafficking to the plasma membrane. We have shown previously that a mutant Ca_V_2.2 channel that is unable to bind Ca_V_β subunits (Ca_V_2.2 W391A) was rapidly degraded (Waithe, D., Ferron, L., Page, K. M., Chaggar, K., and Dolphin, A. C. (2011) *J. Biol. Chem.* 286, 9598–9611). Here we show that, in the absence of Ca_V_β subunits, a construct consisting of the I-II loop of Ca_V_2.2 was directly ubiquitinated and degraded by the proteasome system. Ubiquitination could be prevented by mutation of all 12 lysine residues in the I-II loop to arginines. Including a palmitoylation motif at the N terminus of Ca_V_2.2 I-II loop was insufficient to target it to the plasma membrane in the absence of Ca_V_β subunits even when proteasomal degradation was inhibited with MG132 or ubiquitination was prevented by the lysine-to-arginine mutations. In the presence of Ca_V_β subunit, the palmitoylated Ca_V_2.2 I-II loop was protected from degradation, although oligoubiquitination could still occur, and was efficiently trafficked to the plasma membrane. We propose that targeting to the plasma membrane requires a conformational change in the I-II loop that is induced by binding of the Ca_V_β subunit.

## Introduction

Voltage-gated calcium channels have a crucial role in excitable cells, being involved in neurotransmitter release, gene transcription, muscle contraction, and calcium-induced calcium release from internal stores (2). High voltage-activated calcium channels are heteromultimeric complexes made of a pore-forming Ca_V_α1^3^ subunit (3) along with auxiliary Ca_V_β and Ca_V_α_2_δ subunits (4).

Ca_V_β subunits have two distinct effects on the expression on Ca_V_α1 subunits: first, they influence the biophysical properties of the channels by hyperpolarizing the voltage dependence of activation and increasing the probability of opening (5), and second, they increase the trafficking of the channels to the plasma membrane (6). The binding site for Ca_V_β subunits on Ca_V_α1 was shown to be a highly conserved region of the intracellular loop between domains I and II (I-II loop) of high voltage-activated channels that became known as the α-interaction domain (AID) (7). This conserved motif, consisting of 18 amino acids starting 24 residues after the end of the IS6 transmembrane segment of Ca_V_2.1, was found to bind to all four Ca_V_β subunits (8).

The mechanism by which the Ca_V_β subunit promotes trafficking of the Ca_V_α1 to the plasma membrane is under debate. Apart from Ca_V_β2, which can associate with the plasma membrane either through palmitoylation (9) or by a polybasic region in the case of Ca_V_β2e (10), the Ca_V_β subunits are cytoplasmic proteins. It was first suggested that the Ca_V_α1 I-II loop contained an ER retention motif that was masked in the presence of a Ca_V_β (11), but more recently it has been postulated that the I-II loops contain an acidic ER export signal (12). Fusion proteins containing the Ca_V_α1 I-II loops fused to a transmembrane CD4 sequence were not retained in the ER (13), and evidence suggests that binding of the Ca_V_β subunits to the Ca_V_1.2 and Ca_V_2.2 I-II loop prevented ubiquitination and degradation of these channels (1, 13). Our previous work has shown that a mutant Ca_V_2.2 channel that is unable to bind Ca_V_β subunits (Ca_V_2.2 W391A) was subjected to increased proteasomal degradation relative to the wild-type channel (1).

Efficient degradation by the proteasome requires the substrate to be ubiquitinated. Ubiquitin is a protein of 76 amino acids that is attached by a covalent bond to a lysine residue in the substrate; this then serves as a sorting signal (14). A single ubiquitin molecule can bind, resulting in monoubiquitination. More commonly, as ubiquitin contains seven lysine residues and can itself be ubiquitinated, polyubiquitination occurs. Different types of ubiquitination lead to different fates of the proteins; for example, Lys-48-linked ubiquitination is a signal to target the substrate for degradation via the proteasome (14).

In this study, we show that, in the absence of Ca_V_β subunit, the isolated I-II loop of Ca_V_2.2 is directly ubiquitinated on a lysine residue within the loop and is rapidly degraded. Binding of a Ca_V_β subunit prevents degradation of the ubiquitinated I-II loop but does not prevent oligoubiquitination. Surprisingly, we found that attaching a palmitoylation motif to the N terminus of the I-II loop is insufficient to target it to the plasma membrane in the absence of the Ca_V_β even when protein degradation is inhibited. This suggests that membrane targetting requires the I-II loop to be correctly folded in the presence of the Ca_V_β subunit.

## Results

### 

#### 

##### Trafficking of the Palmitoylated I-II Loop of Ca_V_2.2 in tsA-201 Cells

**FIGURE 1.**
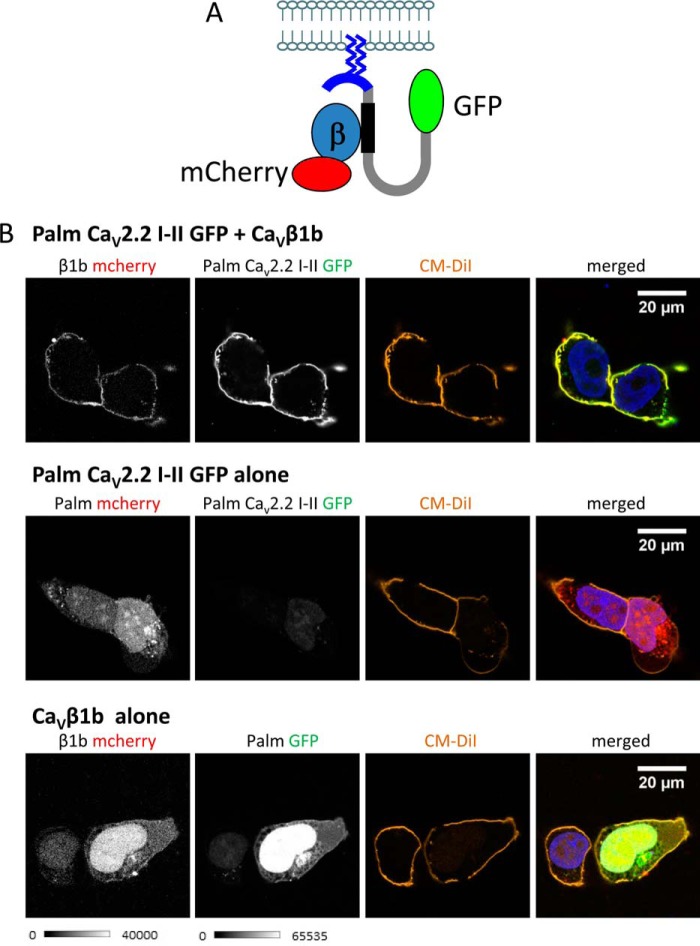
**Palm Ca_V_2.2 I-II-GFP is trafficked to the plasma membrane in the presence of Ca_V_β1b.** *A*, schematic showing the constructs used. The palmitoylation motif, MTLESIMACCL, containing two palmitoylated cysteines and shown in *blue*, was fused to the N terminus of the I-II loop of Ca_V_2.2 (amino acids 356–483). GFP was fused to the C terminus of the I-II loop. Ca_V_β1b, which is fused to mCherry, interacts with the β-binding site on the I-II loop, shown in *black. B*, confocal images of tsA-201 cells co-expressing palm Ca_V_2.2 I-II-GFP (shown in *grayscale*; *top row*, *second panel*) with Ca_V_β1b-mCherry (*top left*) or palm mCherry (in the absence of Ca_V_β; *middle row*). The *bottom row* shows Ca_V_β1b-mCherry (*left*) co-expressed with palm GFP (*second panel*). For each condition, the *third column* shows the cells stained with the plasma membrane marker CM-DiI. Palm Ca_V_2.2 I-II-GFP and Ca_V_β1b-mCherry are only trafficked to the plasma membrane when expressed together. The merged images on the *right* show mCherry in *red*, GFP in *green*, the nuclei stained with DAPI (*blue*), and the plasma membrane stained with CM-DiI (*orange*). The *scale bar* is 20 μm, and the *grayscale calibration bar* is shown below.

To investigate the mechanism by which the Ca_V_β subunit protects Ca_V_2.2 from degradation and is involved in trafficking it to the plasma membrane, a construct was made containing the intracellular I-II loop of Ca_V_2.2 (amino acids 356–483), which is known to interact with the Ca_V_β via the AID (7). A palmitoylation motif, MTLESIMACCL, the first 11 amino acids of the guanine nucleotide-binding protein G_q_ (15), was added to its N terminus (palm Ca_V_2.2 I-II) to target it to the plasma membrane, and a tag, either GFP or HA, was added to the C terminus (Fig. 1*A*).

The expression and localization of the palmitoylated I-II loop of Ca_V_2.2 tagged with GFP (palm Ca_V_2.2 I-II-GFP) was investigated using confocal microscopy. In the presence of Ca_V_β1b tagged with mCherry, palm Ca_V_2.2 I-II-GFP was efficiently trafficked to the plasma membrane as shown by its colocalization with the plasma membrane marker CM-DiI (Fig. 1*B*, *top panel*). In the absence of Ca_V_β subunits, palm Ca_V_2.2 I-II-GFP showed very low expression levels in tsA-201 cells and was not found at the plasma membrane (Fig. 1*B*, *middle panel*) despite the presence of the palmitoylation sequence. Either the palm Ca_V_2.2 I-II-GFP was being expressed at much lower levels in the absence of Ca_V_β1b, or more likely it was being degraded.

When expressed alone, Ca_V_β1b-mCherry was found uniformly distributed throughout the cytoplasm (Fig. 1*B*, *bottom left panel*), but when co-expressed with palm Ca_V_2.2 I-II-GFP, it too was found mainly at the plasma membrane (Fig. 1*B*, *top left panel*). Surprisingly, the palmitoylation motif alone was also insufficient to target mCherry (Fig. 1*B*, *middle row*) or GFP (Fig. 1*B*, *bottom row*) to the plasma membrane; trafficking to the plasma membrane required both palm Ca_V_2.2 I-II and the Ca_V_β subunit (Fig. 1*B*).

**FIGURE 2.**
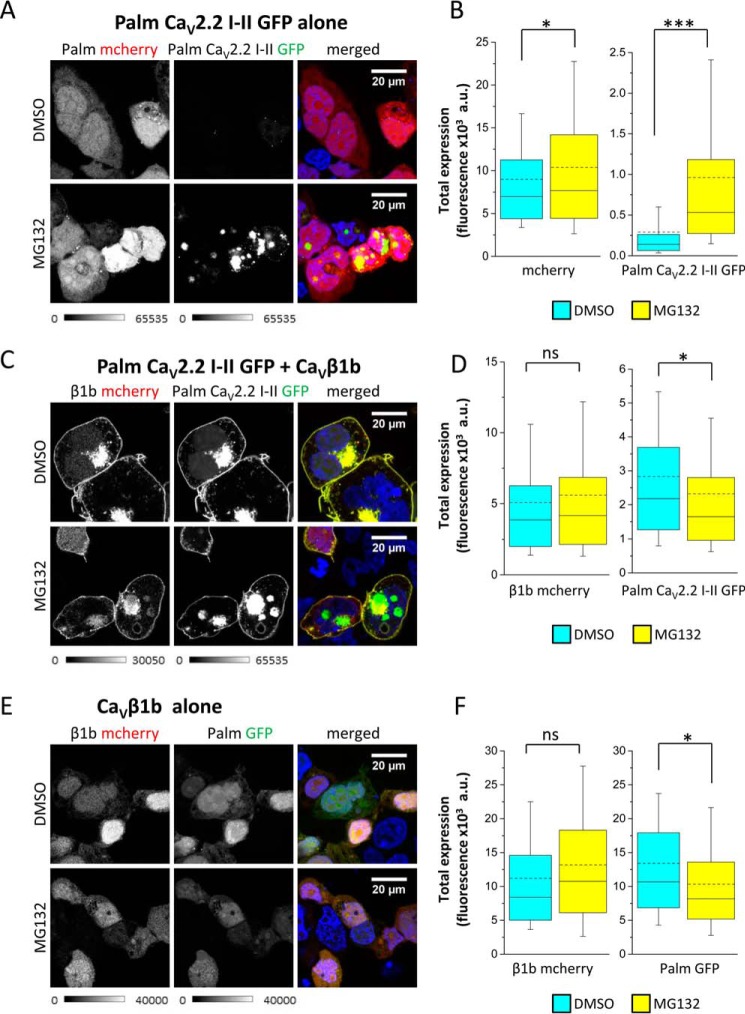
**In the absence of Ca_V_β subunit, palm Ca_V_2.2 I-II-GFP is rapidly degraded.** *A*, representative confocal images of tsA-201 cells co-expressing palm mCherry without Ca_V_β (shown in *grayscale*; *left*) and palm Ca_V_2.2 I-II-GFP (*grayscale*; *middle*) in the absence (*top panels*) and presence (*bottom panels*) of MG132 (4 μm). The panel on the *right* shows the merged image; DAPI was used to visualize the nucleus (*blue*). The *scale bar* is 20 μm, and the *grayscale calibration bar* is shown *below* each set of images. *B*, box and whisker plots showing fluorescence intensity in the absence of MG132 (DMSO control; *cyan bar*; *n* = 221 cells) or the presence of MG132 (*yellow bar*; *n* = 314) of the cells represented in *A*. The *box* shows the interquartile range, *whiskers* range from 10 to 90%, the median is shown as a *solid line*, and the mean is shown as a *dotted line*. GFP fluorescence was measured in all cells that were expressing mCherry. Palm mCherry fluorescence (*left*) was slightly increased in the presence of MG132 (*, *p* = 0.04, Student's *t* test; mean fluorescence ±S.E. of 8977 ± 463 arbitrary units (*a.u.*) in DMSO and 10,379 ± 460 arbitrary units in MG132), whereas palm Ca_V_2.2 I-II-GFP (*right*) fluorescence was significantly increased by MG132 (***, *p* < 0.0001, Student's *t* test; mean ± S.E. of 291 ± 40 (DMSO) and 962 ± 63 arbitrary units (MG132)). *C*, as for *A* but for cells co-expressing palm Ca_V_2.2 I-II-GFP with Ca_V_β1b-mCherry. *D*, box and whisker plots (as in *B*) showing fluorescence intensity of the cells in *C* in the absence (*cyan*; *n* = 243 cells) or the presence (*yellow*; *n* = 244) of MG132. Ca_V_β1b-mCherry (*left*) fluorescence was not significantly altered by MG132 (mean ± S.E. of 5067 ± 264 (DMSO) and 5595 ± 329 arbitrary units (MG132); palm Ca_V_2.2 I-II-GFP (*right*) fluorescence was slightly (*, *p* = 0.02, Student's *t* test) decreased in the presence of MG132 (mean ± S.E. of 2830 ± 145 (DMSO) and 2317 ± 156 arbitrary units (MG132)). *E*, control experiments showing the co-expression of Ca_V_β1b-mCherry and palm GFP as for *A. F*, box and whisker plots (as in *B*) showing fluorescence intensity of the cells in *E*; Ca_V_β1b-mCherry (*left*) or palm GFP (*right*) in the absence (*cyan*; *n* = 86 cells) or presence (*yellow*; *n* = 69) of MG132. Ca_V_β1b-mCherry fluorescence was not significantly different (mean ± S.E. of 11,200 ± 940 (DMSO) and 13,190 ± 1160 arbitrary units (MG132)); palm GFP fluorescence was slightly (*, *p* = 0.03, Student's *t* test) decreased in the presence of MG132 (mean ± S.E. of 13,410 ± 1019 (DMSO) and 10,340 ± 871 arbitrary units (MG132)). *ns*, non-significant.

Whether protein degradation was responsible for reduced palm Ca_V_2.2 I-II-GFP expression in the absence of Ca_V_β was investigated by measuring the levels of fluorescence in the absence and presence of the proteasomal inhibitor MG132 (Fig. 2, *A* and *B*). In the presence of MG132, the levels of palm Ca_V_2.2 I-II-GFP were significantly increased (Fig. 2, *A* and *B*), suggesting that it is usually rapidly degraded by the proteasome. However, it was still not associated with the plasma membrane.

In the presence of Ca_V_β1b tagged with mCherry, palm Ca_V_2.2 I-II-GFP was expressed at increased levels and was trafficked to the plasma membrane (Fig. 2*C*, *middle panel*). There was no increase in fluorescence in the presence of MG132 (Fig. 2*D*), suggesting that Ca_V_β protects palm Ca_V_2.2 I-II-GFP from being degraded. The same result was found using untagged Ca_V_β1b (data not shown). Control experiments showed that expression of either Ca_V_β1b-mCherry or palm GFP alone was not increased by MG132 (Fig. 2, *E* and *F*).

##### Evidence for Multiple Stages of Ubiquitination of the Ca_V_2.2 I-II Loop

**FIGURE 3.**
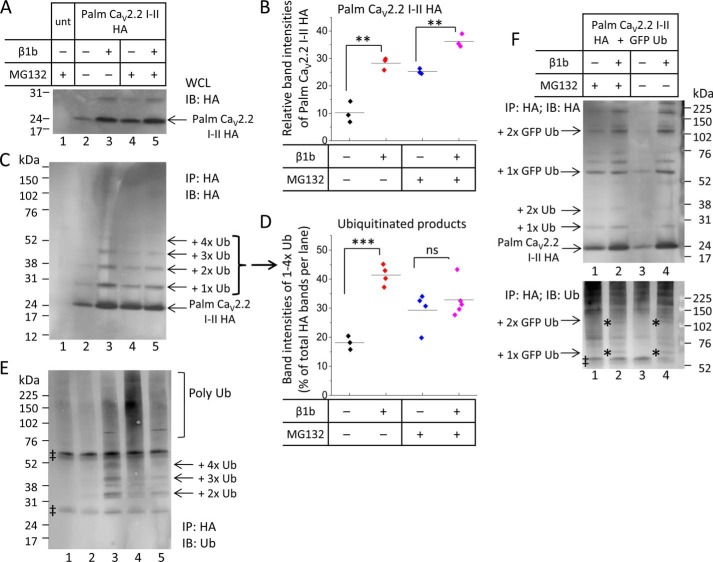
**Palm Ca_V_2.2 I-II-HA is directly ubiquitinated.** *A*, Western blot showing aliquots (30 μg of protein) of WCLs from tsA-201 cells expressing palm Ca_V_2.2 I-II-HA and blotted with rat anti-HA antibody. *Lane 1* shows untransfected (*unt*) cells in the presence of the proteasomal inhibitor MG132 (4 μm). *Lanes 2–5* show cells transfected with palm Ca_V_2.2 I-II-HA in the absence (*lanes 2* and *3*) or presence (*lanes 4* and *5*) of MG132 and the absence (*lanes 2* and *4*) or presence (*lanes 3* and *5*) of Ca_V_β1b. *B*, quantification of band intensities expressed as a scatter plot for palm Ca_V_2.2 I-II-HA from Western blots of WCLs, including the example shown in *A*. Analysis was performed using ImageJ on gels from three separate transfections. The intensity of each band of palm Ca_V_2.2 I-II-HA was measured and expressed as a percentage of total palm Ca_V_2.2 I-II-HA band intensities on each gel. Mean band intensities (shown by *solid lines*) show that, in the absence of MG132 (*A*, *lanes 2* and *3*), Ca_V_β1b causes a significant increase in the amount of palm Ca_V_2.2 I-II-HA (10.22 ± 2.20, *black symbols* in the absence of Ca_V_β; 28.28 ± 1.27, *red symbols* in the presence of Ca_V_β1b; **, *p* = 0.002, Student's *t* test). In the presence of MG132 (*A*, *lanes 4* and *5*), Ca_V_β1b also causes a significant increase in the amount of palm Ca_V_2.2 I-II-HA (25.23 ± 0.565, *blue symbols* in the absence of Ca_V_β; 36.27 ± 1.38, *pink symbols* in the presence of Ca_V_β1b; **, *p* = 0.002, Student's *t* test). *C*, Western blot showing tsA-201 cells immunoprecipitated (*IP*) with rabbit anti-HA antibody and immunoblotted (*IB*) with rat anti-HA antibody. Lanes are the same as those in *A*. A ladder of ubiquitinated I-II loop proteins (indicated by *arrows*) is detected. *D*, quantification of HA-stained Western blots showing the I-II loop bound to one to four ubiquitin molecules as a percentage of total HA protein in each lane. Analysis was performed using ImageJ on gels from three to five separate transfections and includes the example shown in *C*. Band intensities for palm Ca_V_2.2 I-II-HA bound to one to four ubiquitins were combined and expressed as a percentage of total HA proteins per lane. Mean values are represented by *solid lines*. In the presence of Ca_V_β1b, ubiquitinated products make up a larger percentage of HA-detected proteins (mean ± S.E. of 41.38 ± 1.71%, *red symbols*, corresponding to *lane 3* of *C* compared with 18.12 ± 1.34% in the absence of Ca_V_β1b, *black symbols*, corresponding to *lane 2*; ***, *p* = 0.0002, Student's *t* test). In the presence of MG132 (*lanes 4* and *5*), Ca_V_β1b has no significant effect on the percentage of ubiquitinated products detected with the HA antibody (29.28 ± 3.23% in the absence of Ca_V_β1b, *blue symbols* compared with 32.88 ± 2.73% in the presence of Ca_V_β1b, *pink symbols*). *E*, Western blot showing tsA-201 cells immunoprecipitated with rabbit anti-HA antibody and immunoblotted with anti-Ub antibody for the same samples as those shown in *A* and *C*. The ladder of ubiquitinated products detected with the anti-HA antibody (shown in *C*) is also detected with anti-Ub (indicated with *arrows*) along with a smear of polyubiquitinated proteins of high molecular mass. Nonspecific IgG bands detected in all lanes are marked with ‡. *F*, Western blots showing tsA-201 cells transfected with palm Ca_V_2.2 I-II-HA and GFP-Ub, immunoprecipitated with rabbit anti-HA antibody, and immunoblotted with anti-HA (*top*) or anti-Ub (*bottom*) antibodies in the presence (*lanes 1* and *2*) and absence (*lane 3* and *4*) of MG132 and in the absence (*lanes 1* and *3*) and presence (*lanes 2* and *4*) of Ca_V_β1b. GFP-Ub competes with endogenous ubiquitin to bind to palm Ca_V_2.2 I-II-HA to give larger products (*arrows* for +1× or +2× GFP-Ub; also marked with *asterisks* in *bottom* blot). ‡ shows nonspecific IgG bands. *ns*, non-significant.

To investigate the ubiquitination of the Ca_V_2.2 I-II loop in more detail, palmitoylated I-II loop constructs were tagged with hemagglutinin (HA) at the C terminus (palm Ca_V_2.2 I-II-HA) and expressed in tsA-201 cells in the presence or absence of Ca_V_β1b as well as in the presence or absence of the proteasomal inhibitor MG132. The anti-HA antibody identified palm Ca_V_2.2 I-II-HA from whole cell lysates (Fig. 3*A*). Quantification showed that there was significantly less palm Ca_V_2.2 I-II-HA in the absence of Ca_V_β than in the presence of Ca_V_β1b (Fig. 3*B*) in both the absence of MG132 (Fig. 3*B*, compare *columns 1* and *2*, which correspond to Fig. 3*A*, *lanes 2* and *3*) and the presence of MG132 (Fig. 3*B*, *columns 3* and *4*). Ca_V_β, therefore, is likely to protect palm Ca_V_2.2 I-II-HA from being degraded, although an effect on expression cannot be ruled out.

Palmitoylated Ca_V_2.2 I-II-HA proteins were immunoprecipitated from lysates with anti-HA antibody, and Western blotting analysis with both anti-HA and anti-ubiquitin antibodies was carried out. In this experiment, the protein A-Sepharose beads used for the pulldown were limiting; an excess of lysates was added in an attempt to immunoprecipitate equal amounts of palm Ca_V_2.2 I-II loop in the different conditions. Even so, there was less HA-tagged protein present in the absence of both Ca_V_β and MG132 (Fig. 3*C*, *lane 2* compared with *lanes 3–5*).

In the presence of Ca_V_β1b, palm Ca_V_2.2 I-II-HA was found at increased levels (Fig. 3*C*, *lane 3*). The anti-HA antibody identified the palm Ca_V_2.2 I-II-HA at the correct size (18 kDa) but also revealed a ladder of bands at higher molecular mass (Fig. 3*C*). Ubiquitin has a molecular mass of ∼8 kDa. The ladder of bands corresponds to the size expected if one or more endogenous ubiquitins were bound to the Ca_V_2.2 I-II loop. Quantification of band intensity for ubiquitinated I-II loops expressed as a percentage of total protein per lane for HA-stained Western blots showed that the ubiquitinated palm Ca_V_2.2 I-II-HA represented a significantly lower proportion of total I-II loop protein in the absence of Ca_V_β than in the presence of Ca_V_β1b (Fig. 3*D*, *columns 1* and *2*). This difference was lost in the presence of MG132 (Fig. 3*D*, *columns 3* and *4*), suggesting that, in the absence of both Ca_V_β and MG132, ubiquitinated products are rapidly degraded. However, Ca_V_β1b does not appear to prevent the addition of one to four ubiquitins to the I-II loop (Fig. 3*C*, *lanes 3* and *5*).

The ladder of ubiquitinated I-II loop products was also observed using the anti-ubiquitin antibody (Fig. 3*E*), further evidence that these bands represent ubiquitinated I-II loop. The addition of one to four ubiquitins to palm Ca_V_2.2 I-II-HA was more evident in the presence of Ca_V_β1b (Fig. 3, *C* and *E*, *lanes 3* and *5*) than in its absence (*lanes 2* and *4*). It appears that Ca_V_β1b protects against degradation of these oligoubiquitinated products as well as or better than MG132.

There was no evidence of high molecular mass polyubiquitination in the absence of MG132 and Ca_V_β (Fig. 3*E*, *lane 2*), suggesting that, in the absence of the proteasomal inhibitor, the polyubiquitinated Ca_V_2.2 I-II loop was rapidly degraded by the proteasome. In contrast, in the presence of MG132 but the absence of Ca_V_β, palmitoylated Ca_V_2.2 I-II loop degradation was prevented, and there was an accumulation of high molecular mass polyubiquitinated proteins as seen by the smear above 76 kDa observed with the anti-ubiquitin antibody (Fig. 3*E*, *lane 4*). This smear was reduced in the presence of Ca_V_β1b (Fig. 3*E*, *lane 5*), suggesting that Ca_V_β1b protects the Ca_V_2.2 I-II loop from polyubiquitination as well as degradation. The Ca_V_β1b, therefore, appears to allow the addition of one to four ubiquitins to the Ca_V_2.2 I-II but prevents the polyubiquitination that usually leads to degradation.

Further evidence that the ladder of bands shown in Fig. 3, *C* and *E*, represents mono- or oligoubiquitinated products was obtained by including GFP-tagged ubiquitin in the transfection mixture. This competed with endogenous ubiquitin, reducing the density of bands at lower molecular mass (26 and 34 kDa) but producing bands at higher molecular mass (55 and 92 kDa) corresponding to the binding of one or two larger GFP-tagged ubiquitins (Fig. 3*F*). Addition of GFP-tagged ubiquitin was observed both in the presence and absence of Ca_V_β1b (Fig. 3*F*). The greater intensity of bands representing GFP-ubiquitin bound to I-II loop in the presence of Ca_V_β1b (Fig. 3*F*, *lane 4* compared with *lane 3*) suggests that there is less degradation when Ca_V_β1b is present. The smear of polyubiquitin-conjugated proteins visualized by the anti-ubiquitin antibody was much stronger in the presence of the highly expressed GFP-ubiquitin (Fig. 3*F*, *bottom panel*, compared with Fig. 3*E*). The anti-ubiquitin antibody binds to polyubiquitinated proteins much more efficiently than to monoubiquitinated conjugates (16).

##### Ubiquitination Occurs on Lysine Residues within the Ca_V_2.2 I-II Loop

**FIGURE 4.**
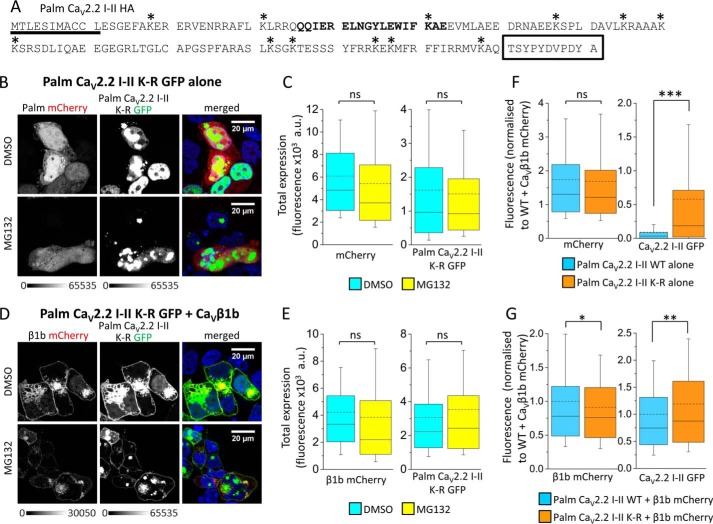
**Mutation of lysine residues in palm Ca_V_2.2 I-II-GFP reduces degradation.** *A*, amino acid sequence of palm Ca_V_2.2 I-II-HA showing the palmitoylation motif (*underlined*) and HA tag (*boxed*). The Ca_V_β-binding domain is shown in *bold*, and lysine residues, which are all mutated to arginines in the K-R mutant, are marked with an *asterisk. B*, representative confocal images of tsA-201 cells co-expressing palm mCherry (without Ca_V_β; *grayscale*; *left*) and palm Ca_V_2.2 I-II K-R-GFP (*grayscale*; *middle*) in the absence (*top panels*) and presence (*bottom panels*) of MG132 (4 μm). The panel on the *right* shows the merged image; DAPI was used to visualize the nucleus (*blue*). The *scale bar* is 20 μm, and the *grayscale calibration bar* is shown below. These images were taken from the same experiment using the same settings as those shown in Fig. 2*A. C*, box and whisker plots showing fluorescence intensity of palm mCherry (*left*) or palm Ca_V_2.2 I-II K-R-GFP (*right*) in the absence of MG132 (DMSO control; *cyan bar*; *n* = 238 cells) or presence of MG132 (*yellow bar*; *n* = 236) of the cells represented in *B*. The *box* shows the range of 25–75%, *whiskers* range from 10 to 90%, the median is shown as a *solid line*, and the mean is shown as a *dotted line*. GFP fluorescence was measured in all cells that were expressing mCherry. MG132 had no significant effect on the mean fluorescence of mCherry or palm Ca_V_2.2 I-II K-R-GFP (mean fluorescence ± S.E.: for mCherry, 6089 ± 272 (DMSO) and 5421 ± 296 arbitrary units (*a.u.*) (MG132); for GFP, 1618 ± 122 (DMSO) and 1507 ± 110 arbitrary units (MG132)). *D*, as for *B* but for cells co-expressing palm Ca_V_2.2 I-II K-R-GFP with Ca_V_β1b-mCherry. *E*, box and whisker plots (as in *C*) showing fluorescence intensity of the cells represented in *D*; Ca_V_β1b-mCherry (*left*) or palm Ca_V_2.2 I-II K-R-GFP (*right*) in the absence of MG132 (*cyan*; *n* = 214 cells) or presence of MG132 (*yellow*; *n* = 226). MG132 had no significant effect on the mean fluorescence of Ca_V_β1b-mCherry (4219 ± 241 (DMSO) and 3846 ± 272 arbitrary units (MG132)) or palm Ca_V_2.2 I-II K-R-GFP (3064 ± 193 (DMSO) and 3523 ± 226 arbitrary units (MG132)). *F* and *G*, normalized confocal data for cells represented in Figs. 2, *A* and *C*, and 4, *B* and *D*, expressed as box and whisker plots (as in *C*). Data have been taken from three independent experiments involving three separate transfections and normalized for mean fluorescence intensity of mCherry and GFP of palm Ca_V_2.2 I-II-GFP WT in the presence of Ca_V_β1b-mCherry (shown in *G*). In the absence of Ca_V_β1b (*F*), mean free mCherry fluorescence (*left*) was not significantly different between WT (1.74 ± 0.07) and the K-R mutant (1.68 ± 0.06), whereas GFP fluorescence (*right*) was significantly lower for WT (0.091 ± 0.011; *light blue*; *n* = 441 cells) than for K-R mutant (0.581 ± 0.040; *orange*; *n* = 552; ***, *p* < 0.0001, Student's *t* test). In the presence of Ca_V_β1b (*G*), mean Ca_V_β1b-mCherry fluorescence was slightly lower for the K-R mutant (0.912 ± 0.027; *n* = 534) than for WT (1.00 ± 0.034; *n* = 518; *, *p* = 0.04, Student's *t* test), whereas GFP fluorescence was slightly higher (1.189 ± 0.045 (K-R) and 1.00 ± 0.040 (WT); **, *p* = 0.002, Student's *t* test). *ns*, non-significant.

Ubiquitination involves the formation of a covalent bond between the C terminus of ubiquitin and the ϵ-amino of a lysine residue on the substrate (17). The palmitoylated Ca_V_2.2 I-II-HA construct contains 12 lysines (Fig. 4*A*, *). To determine whether ubiquitination occurs directly on the I-II loop, all 12 of the lysine residues were mutated to arginines (palm Ca_V_2.2 I-II K-R), and confocal and co-immunoprecipitation experiments were performed on the mutated constructs expressed in tsA-201 cells in the presence or absence of Ca_V_β1b subunits.

Confocal imaging experiments showed that, when expressed alone in tsA-201 cells, the palmitoylated Ca_V_2.2 I-II K-R mutant tagged with GFP (palm Ca_V_2.2 I-II K-R-GFP) was either expressed at higher levels or was less degraded than the wild-type I-II loop (Fig. 4*B* compared with wild type in Fig. 2*A*). However, it was not trafficked to the plasma membrane in the absence of Ca_V_β subunit, again suggesting that the palmitoylation motif alone is not sufficient for trafficking the I-II loop to the plasma membrane. In the presence of MG132, the K-R mutant I-II loop still accumulated in aggregates (Fig. 4*B*) like the wild-type I-II loop, indicating that the mutant is also misfolded in the absence of Ca_V_β. Unlike the wild-type, however, palm Ca_V_2.2 I-II K-R-GFP fluorescence was not increased by MG132 (Fig. 4*C*), suggesting that the K-R mutant undergoes less proteasomal degradation than wild type. In the presence of Ca_V_β1b, both the palmitoylated Ca_V_2.2 I-II K-R-GFP and the mCherry-tagged Ca_V_β1b were found at the plasma membrane (Fig. 4*D*), indicating that Ca_V_β was still able to interact with the I-II loop when all lysine residues were mutated to arginines. Neither palm Ca_V_2.2 I-II K-R-GFP nor Ca_V_β1b-mCherry fluorescence was increased in the presence of MG132 (Fig. 4*E*). The fluorescence intensity of palm Ca_V_2.2 I-II K-R-GFP when expressed without Ca_V_β1b was significantly higher than that of wild-type Ca_V_2.2 I-II-GFP (Fig. 4*F*), whereas in the presence of Ca_V_β1b-mCherry, the difference in GFP fluorescence intensities was much reduced (Fig. 4*G*).

**FIGURE 5.**
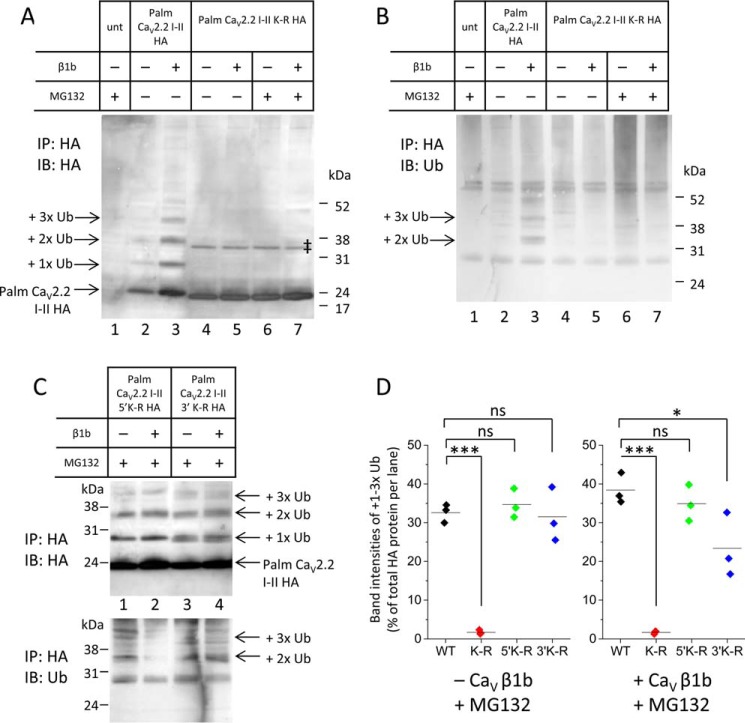
**Ubiquitination of palm Ca_V_2.2 I-II-HA occurs on lysine residues within the loop.** *A* and *B*, Western blots showing tsA-201 cells immunoprecipitated with rabbit anti-HA antibody and immunoblotted with anti-HA (*A*) or anti-Ub (*B*) antibodies. *Lane 1* shows untransfected (*unt*) cells in the presence of the proteasomal inhibitor MG132 (4 μm). *Lanes 4–7* show cells transfected with the mutant palm Ca_V_2.2 I-II K-R-HA in the absence (*lanes 4* and *5*) or presence (*lanes 6* and *7*) of MG132 and in the absence (*lanes 4* and *6*) or presence (*lanes 5* and *7*) of Ca_V_β1b (WT ± Ca_V_β1b is shown in *lanes 2* and *3* for comparison). The ladder of ubiquitinated I-II loop proteins detectable for WT with both antibodies is not detectable for the K-R mutant. The ‡ shown in *A* represents a nonspecific band of ∼35 kDa, possibly a dimer of palm Ca_V_2.2 I-II K-R-HA. *C*, Western blots showing tsA-201 cells expressing two additional mutant I-II loops, either the first four lysine residues mutated to arginines (palm Ca_V_2.2 I-II 5′K-R-HA; *lanes 1* and *2*) or the last eight lysine residues mutated to arginines (palm Ca_V_2.2 I-II 3′K-R-HA; *lanes 3* and *4*) immunoprecipitated (*IP*) with rabbit anti-HA antibody and immunoblotted (*IB*) with anti-HA (*top*) or anti-Ub (*bottom*) antibodies. Ca_V_β1b was transfected as shown, and all samples are in the presence of 4 μm MG132. *D*, quantification of band intensities of ubiquitinated palm Ca_V_2.2 I-II-HA (+1–3× Ub) expressed as a percentage of total HA-detected products per lane. Samples are all in the presence of 4 μm MG132. Band intensities were measured using ImageJ and expressed as scatter plots for WT (*black symbols*; an example of a blot for WT has been shown in Fig. 3*C*) and K-R (*red*), 5′ K-R (*green*), and 3′ K-R (*blue*) mutants. Statistical analysis was carried out using one-way analysis of variance with Bonferroni post hoc test. In the absence of Ca_V_β1b (*left* plot), mean band intensities (*solid lines*) were 32.6 ± 1.36 (WT), 1.70 ± 0.31 (K-R; ***, *p* < 0.0001), 34.70 ± 2.17 (5′ K-R), and 31.52 ± 4.04% (3′ K-R), and in the presence of Ca_V_β1b (*right*), mean band intensities were 38.43 ± 2.30 (WT), 1.68 ± 0.21 (K-R; ***, *p* < 0.0001), 34.91 ± 2.69 (5′ K-R), and 23.36 ± 4.79% (3′ K-R; *, *p* = 0.047). The K-R mutant shows no ubiquitination, whereas both the 5′ K-R and 3′ K-R mutants show significant ubiquitination. *ns*, non-significant.

Western blotting of the palmitoylated Ca_V_2.2 I-II K-R-HA-tagged construct immunoprecipitated with anti-HA antibody showed that the direct binding of ubiquitin to the I-II loop was abolished by mutation of all lysine residues (Fig. 5, *A* and *B*). The ladder of bands corresponding to the size expected for the addition of one or more ubiquitins to the palmitoylated I-II loop (Fig. 5*A*, *lane 3*) was absent for both the anti-HA (Fig. 5*A*, *lanes 4–7*) and anti-ubiquitin antibodies (Fig. 5*B*).

The Ca_V_β subunit is known to interact with the AID within the first half of the I-II loop (7). To identify which part of the I-II loop is involved in ubiquitination, two further K-R mutant HA-tagged constructs were made: one in which the first four lysine residues were mutated to arginines, referred to as 5′ K-R, and the second in which the last eight lysines were mutated to arginines, 3′ K-R. Western blots of the palm Ca_V_2.2 I-II K-R-HA-tagged constructs immunoprecipitated with the anti-HA and blotted with anti-HA and anti-ubiquitin antibodies show that both constructs were able to bind ubiquitin (Fig. 5, *C* and *D*). This indicates either that ubiquitin binds to more than one lysine residue or that it is able to switch to another lysine if the preferred lysine is no longer available.

**FIGURE 6.**
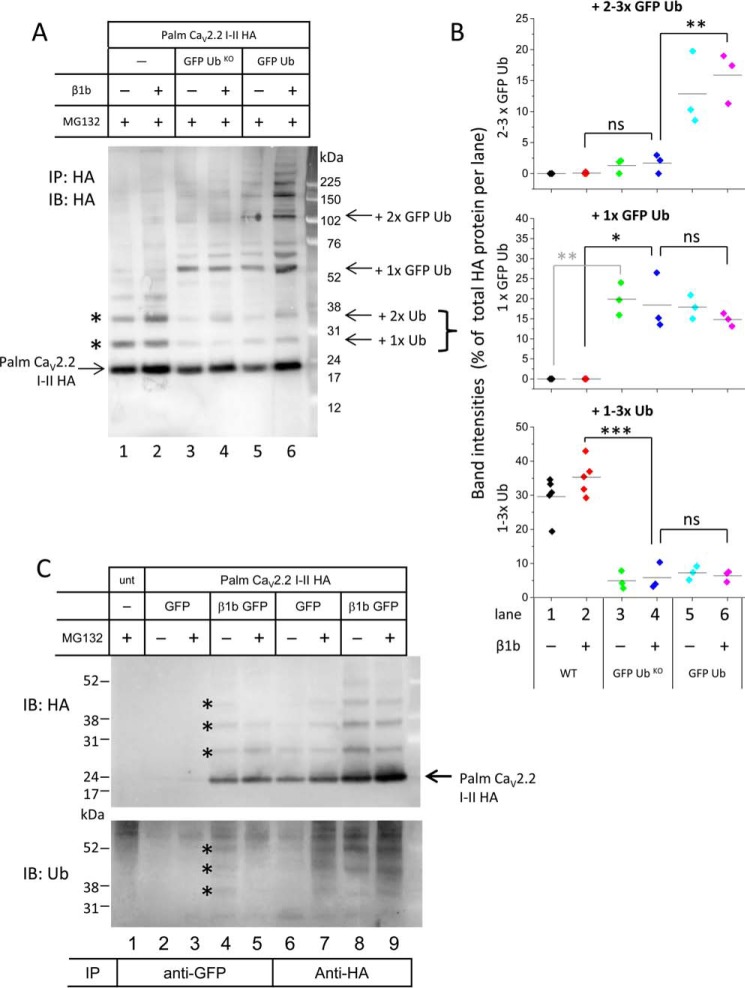
**Oligoubiquitination, occurring on a single lysine within the I-II loop, does not prevent an interaction with Ca_V_β1b-GFP.** *A*, Western blot showing tsA-201 cells expressing WT palm Ca_V_2.2 I-II-HA in the absence (*lanes 1*, 3, and *5*) or presence (*lanes 2*, *4*, and *6*) of Ca_V_β1b and in the presence of GFP-Ub^KO^ (*lanes 3* and *4*) or GFP-Ub (*lanes 5* and *6*) immunoprecipitated (*IP*) and immunoblotted (*IB*) with anti-HA antibody. *Lanes 1* and *2* show palm Ca_V_2.2 I-II-HA bound to endogenous ubiquitins (shown with *asterisks*). GFP-Ub competes with endogenous ubiquitin to give larger products (*arrows* for +1× or +2× GFP-Ub), but only one dominant band is seen when GFP-Ub^KO^ is used. *B*, quantification of band intensities of ubiquitinated palm Ca_V_2.2 I-II-HA expressed as a percentage of total HA-detected products per lane. Samples are all in the presence of 4 μm MG132. Band intensities were measured using ImageJ and expressed as scatter plots and show the percentage of HA-products bound to two or three GFP-tagged ubiquitins (*top*), a single GFP-Ub (*middle*), or one to three endogenous ubiquitins (*bottom*). Lanes are the same as those shown in *A*. In the presence of GFP-Ub^KO^, there is a significant reduction in the amount of endogenous ubiquitins bound to palm Ca_V_2.2 I-II-HA; in the absence of Ca_V_β1b, mean band intensities (*solid lines*) ±S.E. were 29.6 ± 2.68 (WT alone; *black symbols*), 4.92 ± 1.51 (WT + GFP-Ub^KO^; *green*), and 7.24 ± 1.16% (WT + GFP-Ub; *cyan*), and in the presence of Ca_V_β1b, mean band intensities were 35.26 ± 2.35 (WT; *red*), 5.84 ± 2.26 (WT + GFP-Ub^KO^; *blue*; ***, *p* < 0.0001), and 6.39 ± 0.95% (WT + GFP-Ub; *pink*). Statistical analysis was carried out using one-way analysis of variance with Bonferroni post hoc test. For WT alone, there is no measureable amount of GFP-ubiquitin bound (*middle* and *top* plots). Transfecting GFP-Ub^KO^ gives a statistically significant increase in the binding of a single GFP-Ub to palm Ca_V_2.2 I-II-HA in both the absence and presence of Ca_V_β1b (*middle plot*; 19.86 ± 2.34%; *green*; **, *p* = 0.0011 and 18.40 ± 4.06%; *blue*; *, *p* = 0.011), whereas there is no increase in the amount of palm Ca_V_2.2 I-II-HA bound to two or three GFP-Ub moieties (*top*, 1.31 ± 0.66 and 1.70 ± 0.88%). In contrast, in the presence of GFP-Ub, a single GFP-Ub bound (*middle* plot) increases to 17.91 ± 1.7 and 14.78 ± 0.92% in the absence and presence of Ca_V_β1b, respectively, and the addition of two to three GFP-Ub bound (*top*) also increases to 12.87 ± 3.47 and 15.89 ± 2.35% (**, *p* = 0.0048). *C*, Western blots showing tsA-201 cells expressing WT palm Ca_V_2.2 I-II-HA in the presence of free GFP (*lanes 2*, *3*, *6*, and *7*) or Ca_V_β1b tagged with GFP (*lanes 4*, *5*, *8*, and *9*) immunoprecipitated with anti-GFP (*lanes 1–5*) or anti-HA (*lanes 6–9*) and immunoblotted with anti-HA (*top*) or anti-Ub (*bottom*) antibodies. Untransfected (*unt*) cells are shown in *lane 1*. MG132 (4 μm) is included where shown. The size of palm Ca_V_2.2 I-II-HA that is not ubiquitinated is shown with an *arrow. Asterisks* show ubiquitinated products detected with both anti-HA and anti-Ub antibodies. *ns*, non-significant.

To test whether the ladder of bands in Figs. 3 and 5 represents monoubiquitination of multiple lysine residues or oligoubiquitination of a single residue, we used a GFP-tagged mutant ubiquitin construct in which all seven lysine residues were mutated to arginines, GFP-Ub^KO^ (18). This mutant ubiquitin can no longer form polyubiquitinated chains but can still compete with endogenous ubiquitin to monoubiquitinate the substrate. When included in the transfections, a single GFP-Ub^KO^ was found to bind to the palmitoylated Ca_V_2.2 I-II loop (Fig. 6*A*, *lanes 3* and *4*; data quantified in Fig. 6*B*), whereas the ladder of multiple GFP-ubiquitin (Ub) moieties bound was absent (Fig. 6, *A* and *B*, compare *lanes 4* and *lanes 6*). Taken together, these data suggest that only a single lysine residue on the I-II loop is oligoubiquitinated sequentially, but if the preferred lysine has been mutated to arginine, this oligoubiquitination can occur on another available lysine.

##### Ca_V_β1b Does Not Protect Ca_V_2.2 I-II Loop from Oligoubiquitination

Ca_V_β1b protected palm Ca_V_2.2 I-II-GFP from being degraded (Fig. 2, *A–D*). Western blotting, however, showed that palm Ca_V_2.2 I-II-HA was ubiquitinated even when co-transfected with Ca_V_β1b (Fig. 3, *C* and *E*). Although ubiquitination is usually the initial step on the proteasomal degradation pathway, mono- or oligoubiquitination can also lead to different outcomes for the protein (14). We therefore asked whether the I-II loop that was interacting with the Ca_V_β1b was protected from ubiquitination or was also ubiquitinated. To do this, we co-expressed palm Ca_V_2.2 I-II-HA with either GFP-tagged Ca_V_β1b (Ca_V_β1b-GFP) or with GFP (without Ca_V_β) as a control in tsA-201 cells, immunoprecipitated the Ca_V_β with anti-GFP antibody, and immunoblotted with anti-HA and anti-Ub antibodies.

Ca_V_β1b-GFP was able to co-immunoprecipitate non-ubiquitinated palm Ca_V_2.2 I-II-HA (Fig. 6*C*, *lanes 4* and *5*, shown with an *arrow*). In addition, the anti-HA antibody also detected a ladder of bands likely to represent ubiquitinated I-II loop products (Fig. 6*C*, *top*, *lanes 4* and *5*) that were also detected using the anti-Ub antibody (Fig. 6*C*, *bottom*, marked with *asterisks*). Both non-ubiquitinated and ubiquitinated palm Ca_V_2.2 I-II-HA products immunoprecipitated directly with the anti-HA antibody are shown in *lanes 6–9* for comparison. These results, quantified in Fig. 5*D*, could indicate that the I-II loop does not need to be ubiquitinated to interact with Ca_V_β subunits but that Ca_V_β is able to interact with both ubiquitinated and non-ubiquitinated I-II loop. Alternatively, ubiquitin may interact with the Ca_V_2.2 I-II after it has bound to Ca_V_β.

##### Mutation of all Lysine Residues in the I-II Loop Reduces Degradation of Full-length Ca_V_2.2 Channels

**FIGURE 7.**
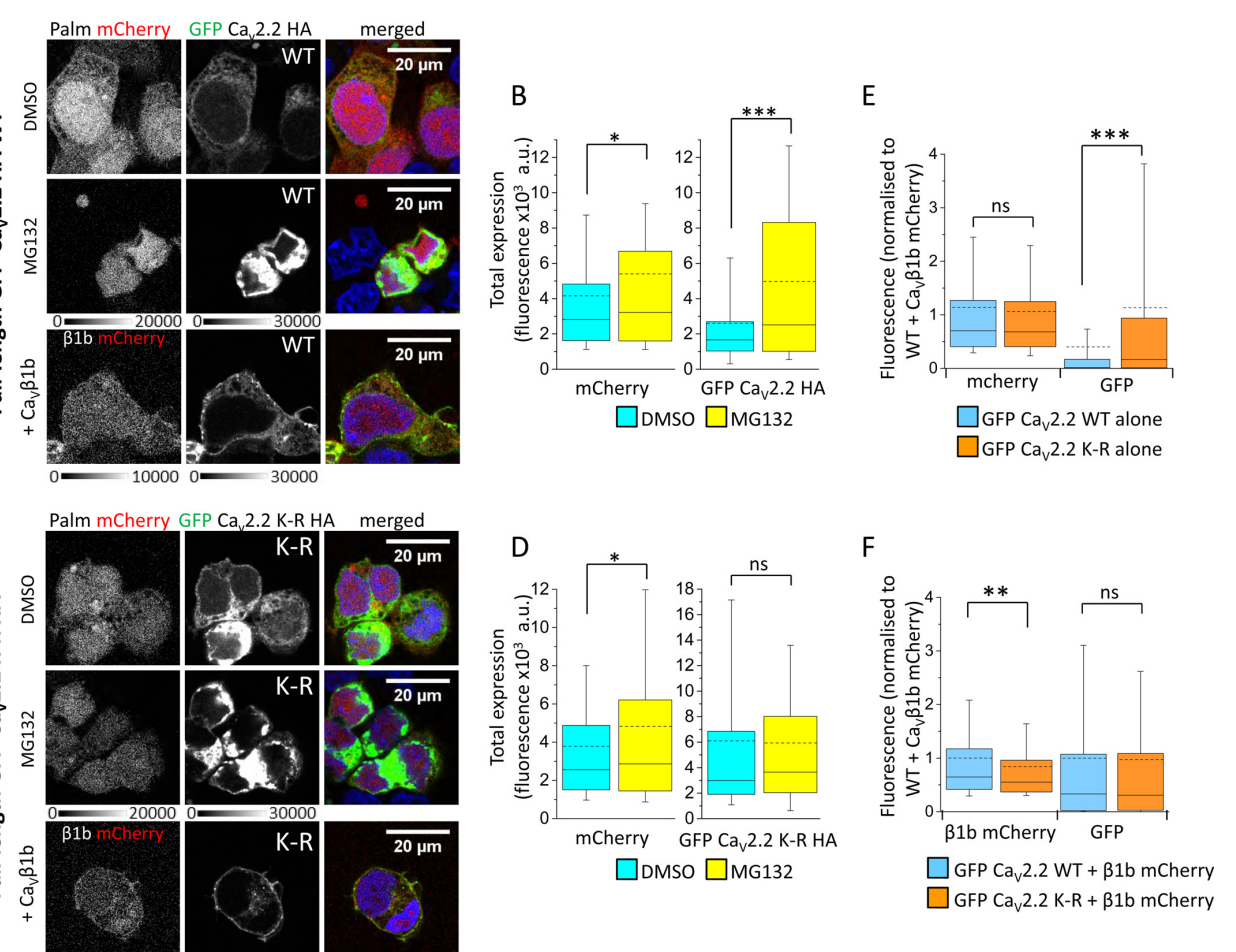
**Full-length Ca_V_2.2 is protected from degradation by mutation of the lysine residues in its I-II loop.** *A*, representative confocal images of tsA-201 cells co-expressing palm mCherry (without Ca_V_β; *left panel*) and full-length GFP-Ca_V_2.2-HA (*middle panel*). The *top row* shows the WT Ca_V_2.2 in the absence of Ca_V_β and MG132, the *middle row* shows palm mCherry and GFP-Ca_V_2.2-HA in the presence of MG132 (4 μm), and the *third row* shows Ca_V_β1b-mCherry with GFP-Ca_V_2.2-HA. The panel on the *right* shows the merged image; DAPI was used to visualize the nucleus (*blue*). The *scale bar* is 20 μm, and the *grayscale calibration bar* is shown below each set of images. *B*, box and whisker plots showing fluorescence intensity in the absence of MG132 (DMSO control; *cyan bar*; *n* = 415 cells) or in the presence of MG132 (*yellow bar*; *n* = 75) of the cells represented in *A*. The *box* shows the range of 25–75%, *whiskers* range from 10 to 90%, the median is shown as a *solid line*, and the mean is shown as a *dotted line*. GFP fluorescence was measured in all cells that were expressing mCherry. Palm mCherry fluorescence (*left*) was slightly increased in the presence of MG132 (*, *p* = 0.046, Student's *t* test) with a mean fluorescence ±S.E. of 4155 ± 210 arbitrary units (*a.u.*) in DMSO *versus* 5403 ± 898 arbitrary units in MG132, whereas palm Ca_V_2.2 I-II-GFP (*right*) fluorescence was significantly increased by MG132 with mean ± S.E. of 2609 ± 153 (DMSO) and 4973 ± 598 arbitrary units (MG132) (***, *p* < 0.0001, Student's *t* test). *C*, as for *A* but for cells expressing Ca_V_2.2 that had all 12 lysine residues in its I-II loop mutated to arginines (K-R). *D*, box and whisker plots (as in *B*) showing fluorescence intensity in the absence of MG132 (DMSO control; *cyan bar*; *n* = 269 cells) or in the presence of MG132 (*yellow bar*; *n* = 138) of the cells represented in *C*. Palm mCherry fluorescence (*left*) was slightly increased in the presence of MG132 (*, *p* = 0.02, Student's *t* test) with mean fluorescence ±S.E. of 3787 ± 233 (DMSO) and 4822 ± 60 arbitrary units (MG132), whereas there was no difference in palm Ca_V_2.2 I-II K-R-GFP fluorescence (*right*; mean ± S.E. of 6098 ± 449 (DMSO) and 5936 ± 520 arbitrary units (MG132)). *E* and *F*, box and whisker plots (as in *B*) of normalized fluorescence intensity of mCherry and GFP for GFP-Ca_V_2.2-HA WT and GFP-Ca_V_2.2 K-R-HA. Data are taken from three independent experiments and normalized for mCherry and GFP fluorescence of WT in the presence of Ca_V_β1b-mCherry (shown in *F*). In the absence of Ca_V_β1b (*E*), mCherry fluorescence (*left*) was not significantly different between WT (*light blue*; *n* = 1678 cells) and the K-R mutant (*orange*; *n* = 1046) with mean normalized fluorescence values ±S.E. of 1.138 ± 0.033 and 1.064 ± 0.037, whereas normalized GFP fluorescence (*right*) was significantly lower for WT than the K-R mutant (***, *p* < 0.0001, Student's *t* test) with mean values of 0.402 ± 0.032 and 1.132 ± 0.069. In the presence of Ca_V_β1b (*F*), although Ca_V_β1b-mCherry expression was slightly lower for K-R mutant (*orange*; *n* = 734; mean, 0.841 ± 0.033) than WT (*light blue*; *n* = 738; mean, 1.00 ± 0.039; **, *p* = 0.0018, Student's *t* test), GFP fluorescence was not significantly different (mean values, 1.00 ± 0.065 for WT and 0.973 ± 0.065 for K-R mutant). *ns*, non-significant.

To determine whether ubiquitination of the I-II loop plays a role in degradation of the full-length channel, the 12 lysine residues in the I-II loop of Ca_V_2.2 (Fig. 4*A*) were mutated to arginines. The wild-type and mutated channels were expressed in tsA-201 cells and examined by confocal imaging. In this experiment, the Ca_V_2.2 α1 subunits contained an extracellular HA tag (6), and GFP was fused to the N terminus to give GFP-Ca_V_2.2-HA (wild type (WT)) or GFP-Ca_V_2.2 K-R-HA (containing 12 lysine-to-arginine mutations). Full-length channels were expressed with auxiliary Ca_V_α_2_δ-1 subunits in the presence or absence of MG132 and the presence or absence of Ca_V_β1b-mCherry (Fig. 7, *A* and *C*). As with the palm Ca_V_2.2 I-II loop, MG132 caused a significant increase in the total GFP-Ca_V_2.2-HA fluorescence measured in the absence of Ca_V_β1b (Fig. 7, *A* and *B*), whereas MG132 had no effect on the GFP fluorescence of GFP-Ca_V_2.2 K-R-HA (Fig. 7, *C* and *D*). In the absence of Ca_V_β1b, total GFP fluorescence intensity was significantly lower for GFP-Ca_V_2.2-HA WT than for GFP-Ca_V_2.2 K-R-HA (Fig. 7*E*). In contrast, in the presence of Ca_V_β1b, GFP fluorescence intensities were not significantly different between the two conditions (Fig. 7*F*). This suggests that the K-R mutations within the I-II loop protect the full-length channel from degradation in the absence of Ca_V_β.

**FIGURE 8.**
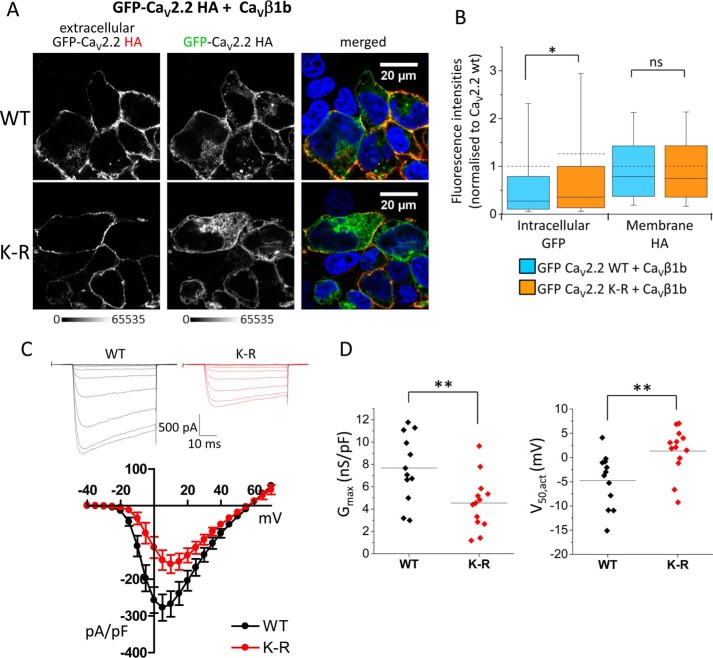
**Full-length GFP-Ca_V_2.2-HA containing 12 lysine-to-arginine mutations in the I-II loop is still trafficked and functionally active in the presence of Ca_V_β1b.** *A*, representative confocal images of tsA-201 cells expressing full-length GFP-Ca_V_2.2-HA with Ca_V_β1b and Ca_V_α_2_δ-1 and showing WT (*top*) and K-R mutant (*bottom*). Cells were non-permeabilized and incubated with rat anti-HA antibody overnight to show extracellular HA staining on the plasma membrane (*left panels*; *grayscale*) to be compared with intracellular GFP fluorescence (*middle panels*; *grayscale*). Merged images are shown on the *right*; DAPI was used to stain the nuclei (*blue*). *Scale bars* are 20 μm, and the *grayscale calibration bar* is shown below. *B*, box and whisker plots showing normalized fluorescence intensities of the images represented in *B* with intracellular GFP (*left*) for WT GFP-Ca_V_2.2-HA (*light blue*; *n* = 1048 cells) and GFP-Ca_V_2.2 K-R-HA (*orange*; *n* = 998 cells) and extracellular HA staining at the plasma membrane (*right*). GFP fluorescence at the plasma membrane (overlapping with membrane HA labeling) was excluded from the analysis to give intracellular GFP rather than total GFP. Data have been taken from three separate experiments involving three transfections. Although intracellular GFP fluorescence may be slightly increased for the K-R mutant compared with WT (mean fluorescent values ±S.E. of 1.000 ± 0.069 for WT and 1.263 ± 0.092 for K-R; *, *p* = 0.022, Student's *t* test), extracellular HA staining is unchanged (mean values, 1.000 ± 0.025 for WT and 1.003 ± 0.029 for K-R). *C*, current-voltage relationships of full-length WT and K-R mutant Ca_V_2.2 channels co-expressed with Ca_V_β1b-GFP and Ca_V_α_2_δ-1 in tsA-201 cells. *Top*, example traces of WT Ca_V_2.2/β1b-GFP/α_2_δ-1 (−40 to +10 mV in 5-mV increments; *black* traces) and K-R Ca_V_2.2/β1b-GFP/α_2_δ-1 currents (−40 to +15 mV in 5-mV increments; *red* traces) recorded from a holding potential of −80 mV. The charge carrier was 1 mm Ba^2+^. Cells expressing wild-type or the K-R mutant channel had the same holding currents of −3 ± 4 pA (*n* = 12) and −3 ± 4 pA (*n* = 13), respectively. The *scale bars* refer to both traces. *Bottom*, mean current density-voltage relationships (pA/pF) were calculated from current amplitude (pA) measurements taken 15 ms into a 50-s depolarizing voltage pulse. *Error bars* represent S.E. *I*_Ba_ at +5 mV was 277 ± 36 pA/pF (*n* = 12) and 148 ± 27 pA/pF (*n* = 13) for WT and K-R Ca_V_2.2, respectively (*p* = 0.0081, Student's *t* test). *D*, characteristic properties of the current density-voltage relationships of WT Ca_V_2.2 and the K-R mutant channel. Values were derived from modified Boltzmann fits of individual current density-voltage plots describing WT and K-R Ca_V_2.2 currents. *Left*, *G*_max_ was 7.7 ± 0.9 nS/pF (mean (*solid line*) ± S.E.; *n* = 12) and 4.5 ± 0.7 nS/pF (*n* = 13) for WT and K-R Ca_V_2.2, respectively (**, *p* = 0.0079, Student's *t* test). *Right*, *V*_50_ values were −4.7 ± 1.6 (*n* = 12) and 1.3 ± 1.3 mV (*n* = 13) for WT and K-R Ca_V_2.2, respectively (**, *p* = 0.0071, Student's *t* test). *ns*, non-significant.

We next wanted to determine whether mutation of the lysines in the I-II loop had any effect on trafficking of the full-length channel. Full-length GFP-Ca_V_2.2-HA (either WT or K-R mutant) was expressed in tsA-201 cells together with Ca_V_β1b and Ca_V_α_2_δ-1. In non-permeabilized conditions, an anti-HA antibody was used to label the channel in the proximity of the plasma membrane (Fig. 8*A*). HA labeling was measured on a line (width of 10 pixels) drawn around the cell, and this was compared with the intracellular GFP fluorescence of the same construct measured within the cell and excluding that at the plasma membrane. Although intracellular GFP fluorescence was slightly higher for GFP-Ca_V_2.2 K-R-HA than for the WT channel in this experiment, the HA fluorescence intensities at the plasma membrane were unchanged (Fig. 8*B*), indicating that the 12 mutations in the I-II loop have little effect on Ca_V_2.2 trafficking.

Electrophysiological examination of Ca_V_2.2-HA channels expressed in tsA-201 cells together with Ca_V_β1b-GFP and Ca_V_α_2_δ-1 showed that the K-R mutant produced a decrease in current density compared with the WT channel (Fig. 8, *C* and *D*). The reduction in current density attributed to the K-R mutation was paralleled by a reduction in whole cell conductance (*G*_max_) through the mutated channel (Fig. 8*D*, *left*). The K-R mutant also had a significantly depolarized *V*_50, act_ (Fig. 8*D*, *right*) compared with WT Ca_V_2.2. As a hyperpolarization in *V*_50, act_ is observed in the presence of the Ca_V_β1b (19), the effect of the K-R mutation on Ca_V_2.2 activation kinetics plausibly reflects a perturbed interaction between the Ca_V_ α1 and Ca_V_ β subunits.

## Discussion

It is well established that the Ca_V_β subunit binds to the Ca_V_α1 channel via the AID in the intracellular I-II loop (7) and that this interaction is required for trafficking the Ca_V_α1 subunit to the plasma membrane (4). Previously we have shown that a mutant Ca_V_2.2 channel that is unable to bind Ca_V_β subunits (Ca_V_2.2 W391A (19)) was rapidly degraded (1). In the present study, we have shown that expression of a construct containing the isolated I-II loop of Ca_V_2.2 with a palmitoylation motif was also rapidly degraded when expressed alone but was efficiently trafficked to the plasma membrane in the presence of the Ca_V_β1b subunit.

The I-II loops of Ca_V_1.3 (20) and other L-type Ca_V_α1 subunits (21) have been shown to be targeted to the plasma membrane even in the absence of Ca_V_β subunits. Trafficking required the presence of a polybasic plasma membrane binding motif consisting of four arginine residues in the distal portion of the Ca_V_1.2 I-II loop (21). In contrast, our experiments show that the palmitoylated I-II loop of Ca_V_2.2 is not targeted to the plasma membrane unless Ca_V_β is present despite the fact that it too has similar clusters of basic amino acids in its distal portion (Fig. 4*A*).

To target the I-II loop to the plasma membrane, we therefore included a palmitoylation motif derived from the G protein Gα_q_. Gα_q_ subunits contain two cysteines close to the N terminus, and palmitoylation of both residues leads to greater stability at the plasma membrane (22). G protein α subunits follow a two-signal model for targeting to the plasma membrane (23). For non-myristoylated G proteins, such as Gα_q_, an α-helical polybasic motif at the N terminus provides the initial signal (24, 25), and this signal, in conjunction with an association with the G protein βγ subunit (Gβγ) (26), allows targeting to the plasma membrane and subsequent palmitoylation (25). Tethering of the Ca_V_β2e subunit to the plasma membrane also requires a polybasic motif at the N terminus (10). However, although the I-II loop of Ca_V_2.2 contains multiple basic amino acids at the N terminus (Fig. 4*A*), this region alone is insufficient to target it to the plasma membrane, even when it is palmitoylated, in the absence of Ca_V_β subunits.

**FIGURE 9.**
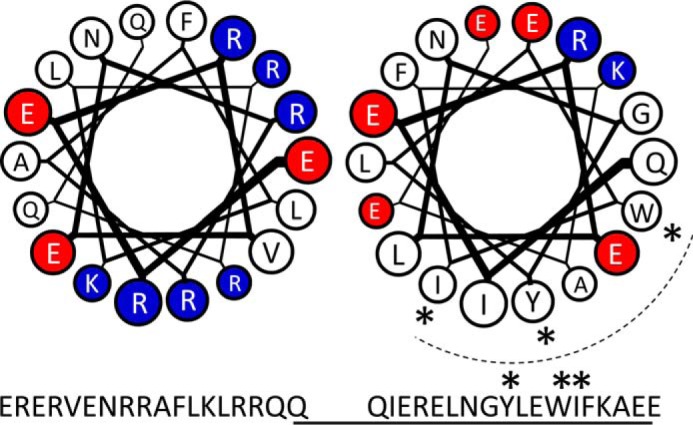
**α-Helical model of the 5′ region of the Ca_V_2.2 I-II loop.** α-Helical wheel models of the 5′ region of the Ca_V_2.2 I-II loop after the IS6 transmembrane domain and immediately preceding the AID (*left*) and the subsequent sequence including the AID (*right*; in the same orientation) show two clusters of basic amino acids along one face of the helix. Basic residues are shown in *blue*, acidic residues are shown in *red*, and the remainder are shown in *white*. Residues involved in the interaction with Ca_V_β subunits lie along the *dotted line*; Trp-391, Ile-392, and Tyr-388, which are critical for this interaction (29), are labeled with *asterisks*.

Binding of Ca_V_β to the AID is thought to induce a dramatic change in secondary structure of the I-II loop (27). In the absence of Ca_V_β, circular dichroism (CD) measurements showed that the AID exists as a random coil (28), whereas in the presence of Ca_V_β, the AID was shown to have an α-helical structure (28, 29). Secondary structure predictions suggested that the entire linker between the last transmembrane domain of domain I (IS6) and the AID could form a continuous α-helix (28), and CD measurements showed that this was the case (30). The helical content was found to be significantly higher for this region of Ca_V_2.2 than that of Ca_V_1.2 (31). It appears that Ca_V_β is able to interact with an unfolded I-II loop via its AID, inducing a conformational change and extending the α-helical structure along the I-II linker toward the IS6 (28). The N terminus of the I-II loop contains a number of basic amino acids (see Fig. 4*A*). Fig. 9 shows two helical wheel diagrams of the region of the I-II linker upstream of the AID (*left*) and including the AID (*right*); formation of an α-helix would allow two clusters of basic amino acids to align along one side and form a polybasic region. This may act as a trafficking signal to direct the construct to the plasma membrane and promote stable dipalmitoylation. Interestingly, the Trp-391 residue, which is critical for the interaction between the I-II loop and the Ca_V_β subunit, is located along the same face of the helix although beyond the polybasic regions (Fig. 9).

Alternatively, formation of an α-helix may allow another binding partner to interact with the I-II loop and direct it to the plasma membrane. The I-II linkers of Ca_V_2.1 (32) and Ca_V_2.2 (33) were found to interact directly with Gβγ. We have previously shown that Ca_V_β and Gβγ subunits bind at the same time and that Ca_V_β must be present for the Gβγ subunit to induce voltage-dependent modulation of Ca_V_2.2 (5). Binding of Gβγ subunits was found to be dependent on the formation of a rigid IS6-AID linker induced by the binding of Ca_V_β (34). The G protein Gα_q_ has been shown to require an association with Gβγ before it can be palmitoylated and stabilized at the plasma membrane (26). To examine whether Gβγ subunits were involved in the trafficking of the I-II loop constructs, we included a minigene derived from the C terminus of β-adrenergic receptor kinase (5) to bind and remove free Gβγ subunits. Confocal experiments showed that transfection of β-adrenergic receptor kinase had no effect on the trafficking of palm Ca_V_2.2 I-II in the presence of Ca_V_β1b,^4^ providing no evidence that Gβγ binding is involved.

In the absence of Ca_V_β, the I-II loop remains in a misfolded state. The Ca_V_β subunit is able to act as a molecular chaperone to induce correct folding of the I-II loop of the Ca_V_α1 subunit. Misfolded proteins are more likely to aggregate as they have exposed hydrophobic domains, and a key role of the chaperone is to interact co-translationally as the nascent protein emerges from the ribosome and prevent misfolding and aggregation (35). In the absence of the chaperone, misfolded proteins are rapidly targeted to the ubiquitin-proteasome system for degradation.

Substrates for the ubiquitin-proteasome system are tagged with ubiquitin; addition of ubiquitin moieties is thought to occur sequentially, one at a time, with the rate-limiting step being the addition of the first ubiquitin (36). It has previously been suggested that a chain of four ubiquitins is the minimum signal for targeting the substrate to the proteasome (37), but more recently it has been shown that only a single ubiquitin is needed for small proteins (20–150 amino acids) (38). Ubiquitination also has other functions, however, such as protein trafficking (14), endocytosis, and signaling (39), which are independent of the proteasome. Our data show that the palmitoylated I-II loop carries up to four ubiquitins (Fig. 3) even in the presence of the Ca_V_β subunit and the proteasomal inhibitor MG132; conditions in which the protein is not degraded. Relevant to this, a mass spectrometry study has shown that in almost 50% of cases, ubiquitination of proteins has a non-proteasomal function (40).

In the absence of Ca_V_β, the I-II loop was rapidly degraded as shown in Fig. 2. It has been shown that nascent proteins can be ubiquitinated co-translationally and targeted for degradation before translation has finished (41). Ubiquitination does not necessarily lead to degradation of newly synthesized ubiquitinated proteins; if correct folding does occur with the help of a chaperone, the ubiquitins may be removed by the action of deubiquitinating enzymes (42). In this regard, binding of the Ca_V_β subunit to the ubiquitinated I-II loop clearly rescues it from degradation, although our evidence does not indicate it promotes deubiquitination. By contrast, it is possible that oligoubiquitination is functionally important for the effect of Ca_V_β because the full-length Ca_V_2.2 K-R mutant showed reduced functional expression, possibly reflecting reduced interaction with the Ca_V_β subunit.

Mutation of the 12 lysine residues in the I-II loop of the full-length Ca_V_2.2 channel protected the channel from degradation. The full-length K-R mutant channel still contains multiple internal lysine residues that were not mutated and were therefore available as ubiquitination sites. However, the fact that the K-R channel was less degraded than WT shows that the 12 lysines within the I-II loop have a critical role in ubiquitination and degradation.

Trafficking of the palmitoylated Ca_V_2.2 I-II loop to the plasma membrane required the presence of the palmitoylation signal, the I-II loop, and the Ca_V_β subunit. Although the palmitoylated K-R mutant I-II loop was no longer ubiquitinated and degraded, it also was not trafficked to the plasma membrane in the absence of the Ca_V_β subunit; trafficking was restored when Ca_V_β was also co-expressed. It is possible that correct folding, which is only achieved in the presence of the Ca_V_β subunit, exposes a trafficking signal. Typical trafficking signals have been identified as short linear amino acid sequences, but it has been shown that export of the Kir2.1 channel from the Golgi to the plasma membrane requires the correctly folded N and C termini of the channel (43). The trafficking signal was only exposed when two separate domains were folded correctly in the tertiary structure of the channel.

In conclusion, our experiments indicate that, in the absence of Ca_V_β subunit, the I-II loop of Ca_V_2.2 is directly ubiquitinated and rapidly degraded by the proteasome system. When Ca_V_β is present, it is able to bind to the I-II loop even if the loop is already ubiquitinated. Binding appears to induce a conformational change and the formation of an α-helix in the I-II loop, thereby allowing the construct to associate with the plasma membrane.

## Experimental Procedures

### 

#### 

##### Molecular Biology

The calcium channels used were rabbit Ca_V_2.2 (GenBank Accession number D14157), rat Ca_V_α_2_δ-1 (M86621), and rat Ca_V_β1b (X61394), all expressed in the pMT2 vector. The green fluorescent protein mut3bGFP (GFP) (44) or mCherry was fused to the C terminus of Ca_V_β1b. GFP-Ub and GFP-Ub^KO^ (18) were obtained from Addgene. The first intracellular loop of Ca_V_2.2 (amino acids 356–483, beginning ESGEF … and ending … KAQ) had a palmitoylation sequence, MTLESIMACCL, added to the N terminus and an HA tag (TSYPYDVPDYA) added to the C terminus to give a construct termed palm Ca_V_2.2 I-II-HA in this study. Further constructs were made where the HA tag was substituted with GFP or mCherry. The mutant palm Ca_V_2.2 I-II K-R-HA was made by mutating all 12 lysine residues in the I-II loop to arginines, and two further constructs were made by mutating either the first four (palm Ca_V_2.2 I-II 5′ K-R-HA) or the last eight (palm Ca_V_2.2 I-II 3′ K-R-HA) lysines to arginines. The I-II loop containing the 12 lysine-to-arginine substitutions was inserted into the full-length Ca_V_2.2, which also contained an extracellular HA tag (6) and GFP fused to the N terminus to give GFP-Ca_V_2.2 K-R-HA. The sequences of all constructs were confirmed by DNA sequencing.

##### Cell Culture and Transfection

tsA-201 cells (European Collection of Authenticated Cell Cultures (ECACC)) were cultured in Dulbecco's modified Eagle's medium in the presence of 10% fetal bovine serum, penicillin, streptomycin, and 2% GlutaMAX (Invitrogen). For immunocytochemistry and co-immunoprecipitation experiments, transfections were carried out using PolyJet (SignaGen) according to the manufacturer's instructions using equal ratios of constructs unless otherwise stated. When included, the protease inhibitor MG132 (Calbiochem) was added at a concentration of 4 μm 24 h after transfection, and the cells were harvested or fixed a further 16–18 h afterward. At the concentration of MG132 used (4 μm) and the time of incubation (16–18 h), MG132 was found to be effective at inhibiting proteasomal degradation without being detrimental to cell survival. Total protein yields from transfections in the absence and presence of MG132 were measured (for Western blotting and co-immunoprecipitation analysis) and found to be similar. For electrophysiological experiments, transfections were performed using FuGENE 6 (Promega) with GFP-Ca_V_2.2-HA, Ca_V_α_2_δ-1, and Ca_V_β1b subunits in a ratio of 3:2:2.

##### Immunocytochemistry

Cells were transfected on polylysine-coated coverslips and fixed with 4% paraformaldehyde in Tris-buffered saline (TBS; 20 mm Tris, 150 mm NaCl, pH 7.4) for 5 min. 4′,6-Diamidino-2′-phenylindole dihydrochloride (DAPI) was used to stain the nuclei. When used, the plasma membrane stain CM-DiI (ThermoFisher Scientific) was used at a dilution of 1:200 for 20 min at room temperature. Coverslips were mounted in VectaShield (Vector Laboratories). When anti-HA staining was used, cells were incubated with blocking buffer (20% goat serum, 4% BSA in TBS) for 1 h at room temperature before being incubated with rat anti-HA (Roche Applied Science) diluted 1:200 in 0.5× blocking buffer at 4 °C overnight. After washing, samples were incubated with secondary antibody anti-rat Alexa Fluor 594 at a dilution of 1:500 for 1 h at room temperature before being stained with DAPI and mounted. Samples were viewed on an LSM 780 confocal microscope (Zeiss) using a 63×/1.4 numerical aperture oil immersion objective in 16-bit mode. The tile function (3 × 3 tiles; each tile consisting of 1024 × 1024 pixels) was used, and every transfected cell within the image was analyzed to remove collection bias. Confocal optical sections were 1 μm, and acquisition settings were kept constant. Images were analyzed using NIH ImageJ. Images that were analyzed were not saturated.

##### Western Blotting Analysis

Transfected tsA-201 cells were harvested in phosphate-buffered saline (PBS) containing protease inhibitors (Complete tablet from Roche Applied Science). Cells were lysed by sonication for 10 s with 1% Igepal in PBS in the presence of protease inhibitors followed by incubation on ice for 30 min, and whole cell lysates (WCLs) were collected after centrifugation (14,000 × *g* for 30 min at 4 °C). Samples were incubated for 15 min at 55 °C with 100 mm dithiothreitol and 2× Laemmli sample buffer, and proteins were separated by SDS-PAGE on 4–12% Bis-Tris gels and then transferred to polyvinylidene fluoride membranes. Membranes were incubated in blocking buffer (10 mm Tris, pH 7.4, 500 mm NaCl, 0.5% Igepal, 3% BSA) for 1 h followed by incubation with the primary antibody. The following primary antibodies were used: rat anti-HA (Roche Applied Science) at 1:1000 overnight, mouse anti-ubiquitin (P4D1, Santa Cruz Biotechnology) at 1:500 overnight, and mouse anti-GFP (Roche Applied Science) at 1:1000 for 1 h, all at 4 °C. The appropriate secondary antibodies coupled to horseradish peroxidase were incubated at a dilution of 1:2000 for 1 h at room temperature. Proteins were detected using the enhanced ECL Plus reagent (GE Healthcare) on a Typhoon 9410 scanner (GE Healthcare). Quantification of Western blotting was carried out using the gel analysis tool of ImageJ. A rectangular section was placed over the entire lane, and each selected lane was plotted as a line profile. The peak band intensities above background were measured. This allows the changes in background along the length of the lane to be taken into account and accurately subtracted.

##### Immunoprecipitation

The total protein concentration was determined (Bradford assay, Bio-Rad) for each sample, and 1 mg of WCL was precleared on 50 μg of protein A-Sepharose (GE Healthcare) for 2 h at 4 °C. Sepharose beads were discarded, and supernatants were incubated with rabbit anti-HA (Sigma) or rabbit anti-GFP (Clontech) at a dilution of 1:200 overnight at 4 °C. Immunoprecipitated proteins were captured on 60 μg of protein A-Sepharose beads for 2 h at 4 °C. Beads were washed five times with PBS containing 0.1% Igepal and incubated for 15 min at 55 °C with 100 mm dithiothreitol and 2× Laemmli sample buffer. Eluted proteins were then resolved by SDS-PAGE.

##### Electrophysiology

Whole cell voltage clamp recordings were performed on tsA-201 cells at room temperature (20–24 °C). Single cells were clamped using an Axopatch 200B patch clamp amplifier (Axon instruments). Borosilicate glass patch pipettes were filled with a solution containing 140 mm cesium aspartate, 5 mm EGTA, 2 mm MgCl_2_, 0.1 mm CaCl_2_, 2 mm K_2_ATP, and 10 mm HEPES. CsOH was added to achieve pH 7.2. The external solution contained 150 mm tetraethylammonium bromide, 3 mm KCl, 1 mm NaHCO_3_, 1 mm MgCl_2_, 10 mm HEPES, 4 mm glucose, and 1 mm BaCl_2_. pH was adjusted to 7.4 with Tris base. Current density-voltage relationships were fitted with a modified Boltzmann equation as follows: *I* = *G*_max_ × (*V* − *V*_rev_)/(1 + exp(−(*V* − *V*_50, act_)/*k*)) where *I* is the current density (in pA/pF), *G*_max_ is the maximum conductance (in nS/pF), *V*_rev_ is the reversal potential, *V*_50, act_ is the midpoint voltage for current activation, and *k* is the slope factor.

## Author Contributions

A. C. D. and K. M. P. designed the experiments. K. M. P. made the cDNA constructs and performed all confocal microscopy, immunoprecipitation, and Western blotting experiments. K. M. P. analyzed data. S. W. R. designed, performed, and analyzed electrophysiology experiments. K. M. P. and A. C. D. wrote the manuscript.

## Footnotes

4 K. M. Page, unpublished observations.

## Footnotes

3 The abbreviations used are:

Ca_V_: voltage-gated calcium
AID: α-interaction domain
Ub: ubiquitin
palm: palmitoylated
Bis-Tris: 2-[bis(2-hydroxyethyl)amino]-2-(hydroxymethyl)propane-1,3-diol
pF: picofarad
nS: nanosiemens
ER: endoplasmic reticulum
WCL: whole cell lysate.
